# Regional intra-arterial infusion chemotherapy combined with deep hyperthermia and targeted therapy followed by radiotherapy for recurrence with intestinal obstruction after radical resection of rectal cancer in an elderly patient: a case report of individualized treatment

**DOI:** 10.3389/fonc.2026.1832384

**Published:** 2026-05-25

**Authors:** Guangsheng Zhao, Shunhang Yin, Xinyu Zhou, Fang Xu, Licai Xu, Qiushi Wang, Jun Zhou, Lei Zhang, Song Liu

**Affiliations:** 1 Cancer Interventional Center, Affiliated Zhongshan Hospital of Dalian University, Dalian, Liaoning, China; 2 Anorectal Surgery, Affiliated Zhongshan Hospital of Dalian University, Dalian, Liaoning, China; 3 Dalian Medical University, Dalian, Liaoning, China; 4 Interventional Medicine Center, Linyi Cancer Hospital, Linyi, Shandong, China

**Keywords:** deep hyperthermia, intestinal obstruction, palliative radiotherapy, rectal cancer, regional intra-arterial infusion chemotherapy

## Abstract

The local recurrence rate after radical resection of rectal cancer is approximately 5%-20%, and can increase to 20%-35% in elderly patients who do not receive radiotherapy or chemotherapy. Recurrence is frequently complicated by intestinal obstruction, with a median survival of only 6–7 months. Herein, we report a case of an elderly patient who developed local recurrence with complete intestinal obstruction 4 months after radical resection of rectal cancer. After obstruction relief achieved by intestinal stent placement, a comprehensive treatment strategy was implemented based on genetic testing and multidisciplinary team discussion, consisting of regional intra-arterial infusion chemotherapy combined with deep hyperthermia and bevacizumab, followed by sequential radiotherapy. The patient ultimately achieved complete remission and long-term stable disease.

## Introduction

Global cancer statistics for 2024 indicate that colorectal cancer ranks second in incidence and fourth in mortality, with approximately 4 million new cases of rectal cancer annually (1). The incidence of rectal cancer in China is substantially higher than the global average, representing the second most common gastrointestinal malignancy, a trend associated with dietary westernization, low screening rates, and other factors (2). Elderly patients often fail to complete standard chemoradiotherapy due to poor tolerance, leading to postoperative recurrence rates of 20%-35%. Such recurrences are frequently complicated by intestinal obstruction, with a median survival of only weeks to months (3). Regional intra-arterial infusion chemotherapy (RIAC) is an important locoregional treatment for colorectal cancer; It shows promising potential in locally advanced disease by markedly increasing local drug concentrations while reducing systemic toxicity (4, 5). Hyperthermia exerts synergistic antitumor effects through direct cytotoxicity and indirect immunomodulation; It enhances chemoradiosensitivity, reduces dose-dependent toxicities, and remodels the tumor microenvironment to reverse immunosuppression (6–8). Clinical evidence supports that hyperthermia improves the efficacy of chemoradiotherapy in locally advanced rectal cancer with a manageable safety profile (9). For elderly patients, Chinese expert consensus recommends individualized strategies based on comprehensive assessment of tumor features and frailty. Meta-analyses further confirm that multimodality therapy can be safely administered in this population with appropriate patient selection (10). This case report was prepared in accordance with the CAse REport (CARE) guidelines.

Herein, we present the case of an 81-year-old male patient who developed local recurrence with complete intestinal obstruction four months after radical resection of rectal cancer. Following intestinal stenting to relieve obstruction, a comprehensive treatment strategy was administered, including RIAC combined with deep hyperthermia and bevacizumab, followed by sequential palliative radiotherapy. The patient ultimately achieved complete remission with durable long-term disease control. No severe adverse events were observed during treatment, and follow-up revealed favorable quality of life and preserved performance status.

## Patient information

An 81-year-old male presented to our emergency department with a 1-week history of cessation of flatus and defecation, accompanied by abdominal pain and distension. According to the surgical and pathological records from another hospital, the patient underwent low anterior resection with total mesorectal excision (TME) four months previously. Postoperative pathology revealed poorly differentiated adenocarcinoma, with a pathological stage of pT3N0M0 and R0 resection. Although this stage warranted postoperative adjuvant chemotherapy, no adjuvant treatment was administered owing to the patient’s advanced age (81 years) and concerns from both the patient and the family regarding chemotherapy-related toxicities. On current admission, the patient’s carcinoembryonic antigen (CEA) level was 42.97 ng/mL, and the Eastern Cooperative Oncology Group (ECOG) performance status score was 3. Written informed consent was obtained from the patient for publication of this case report and any accompanying images, in accordance with the CARE guidelines.

## Clinical findings

The patient presented with typical symptoms of complete intestinal obstruction, including abdominal distension, tenderness, and absence of bowel sounds. Due to the obstruction and high tumor burden, his overall condition was poor.

### Diagnostic assessment

**Figure 1:**
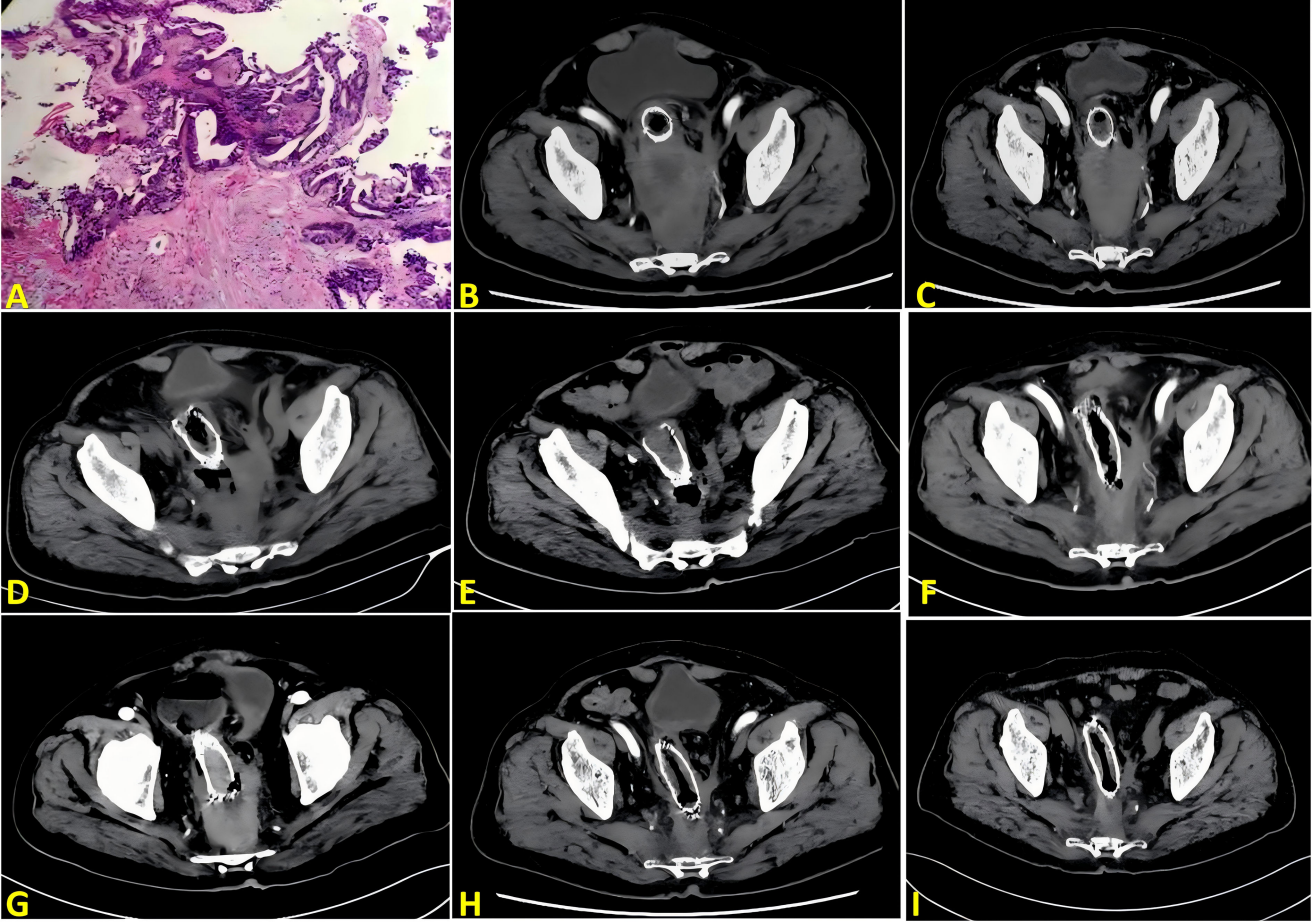
**(A)** Colonoscopic biopsy pathology revealed moderately to poorly differentiated adenocarcinoma. **(B)** Contrast-enhanced abdominal CT demonstrated a rectal space-occupying lesion with marked enhancement, measuring 115 mm × 70 mm, invading the bladder with fistula formation, and left hydroureter with hydronephrosis. **(C)** CT performed 3 weeks after the first interventional procedure showed reduction in tumor size. **(D)** CT performed 1 week after the third interventional procedure revealed further tumor shrinkage, marked hypodensity, and cavitary necrosis surrounding the stent. **(E)** Three weeks after the third interventional procedure, the necrotic portion of the tumor was partially absorbed, with further reduction in tumor size and residual necrosis visible internally. **(F)** Three months after the first interventional procedure, the tumor had markedly decreased in size compared with previous images. **(G)** Four months after the first interventional procedure, the patient developed recurrent intestinal obstruction; CT showed tumor in growth within the intestinal stent and severe dilatation of the distal colon. **(H)** One month after palliative radiotherapy, the tumor had significantly regressed and disappeared, with no enhancement observed in residual lesions. **(I)** One year after the first interventional procedure, CT demonstrated a patent intestinal stent with no evidence of space-occupying lesions or abnormal enhancement at the margins.

Colonoscopy revealed a 2–3 cm irregular elevated lesion located 4 cm from the dentate line, consistent with postoperative local recurrence. The lumen was severely stenotic, preventing passage of the endoscope. Pathological examination of the biopsy specimen confirmed moderately to poorly differentiated adenocarcinoma (**Figure 1A**), consistent with the previous postoperative pathology. Immunohistochemistry showed: CK7(-), CK20 (partial+), Ki67 (90%+), P53 (mutant type), HER-2(0), PD-L1 (SP263, CPS = 5), MLH1(+), MSH2(+), MSH6(+), PMS2(+), S-100(+), CD31(-), D2-40(-), CKpan(+), and panTRK(-).

Contrast-enhanced abdominal CT demonstrated localized thickening of the rectal wall with luminal narrowing and severe colonic dilatation. The tumor invaded the bladder and left ureter, accompanied by bladder fistula formation, left hydroureter, and hydronephrosis. Imaging findings were consistent with postoperative recurrence of rectal cancer complicated by intestinal obstruction and adjacent organ invasion.

Tumor genetic testing revealed the following: Gene mutations-TP53 exon5 c.524G>A (p.Arg175His) missense mutation (allele frequency 38.99%); APC p.Ser367fs frameshift mutation, p.Glu468 nonsense mutation, and p.Arg1450* nonsense mutation. Wild-type-KRAS, NRAS, BRAF. Germline variants-No pathogenic mutations detected in BRCA1/2 or MMR genes. MSI/MMR status-Microsatellite stable (MSS)/proficient mismatch repair (pMMR). PD-L1 expression-Negative (TPS<1%, CPS<1). HLA typing-No HLA class I loss of heterozygosity detected. Sensitive agents-Chemotherapy: oxaliplatin, fluorouracil, irinotecan; Targeted therapy: cetuximab, bevacizumab.

### Therapeutic intervention

**Figure 2:**
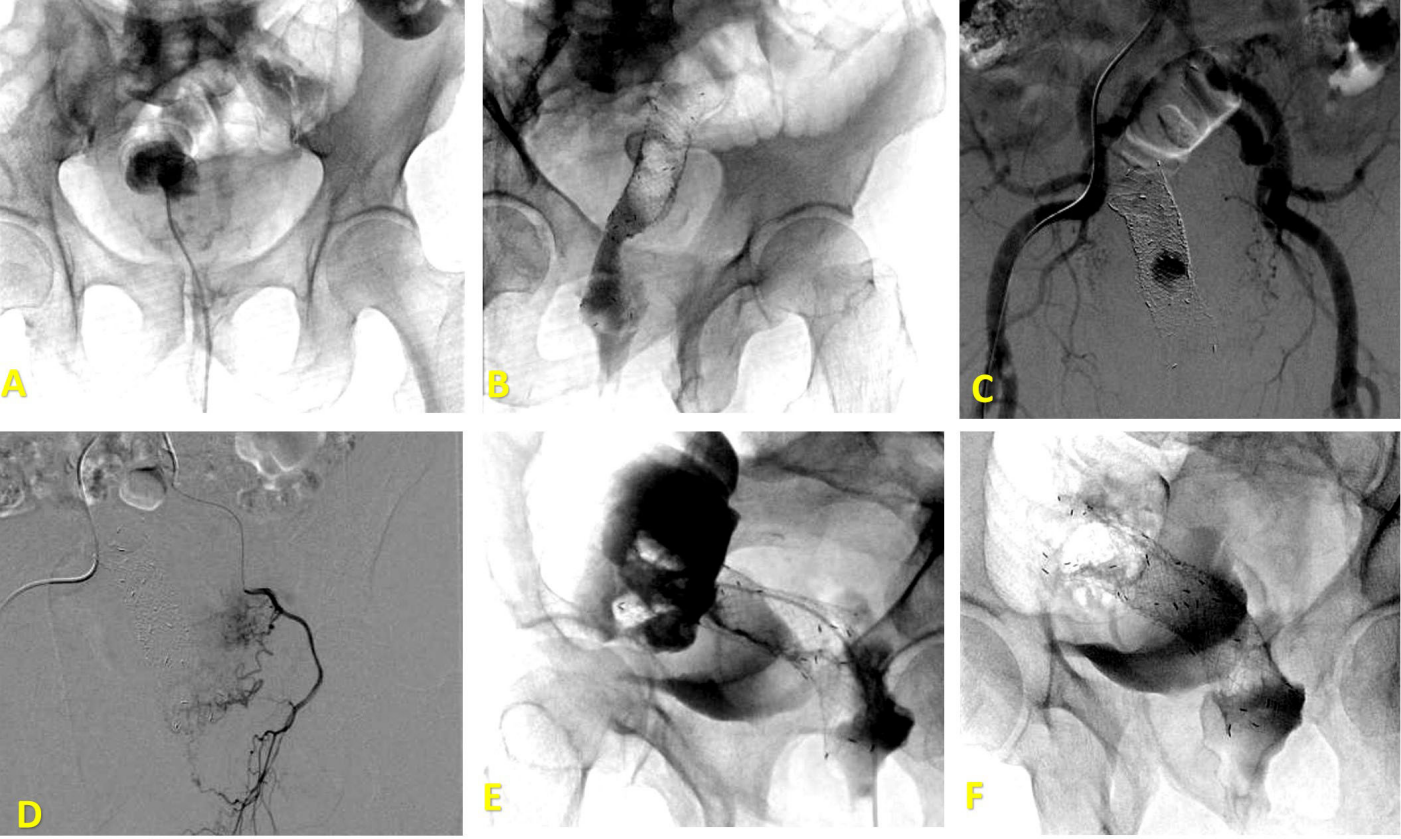
**(A)** Rigid rectal wall with marked dilatation of the distal intestine and contrast agent retention distal to the lesion. **(B)** After intestinal stent placement, contrast agent and intestinal gas passed smoothly through the stent and were excreted. **(C)** Arterial angiography revealed tumor staining surrounding the rectal stent, predominantly on the left side. **(D)** Following superselective catheterization of the tumor-feeding artery with a microcatheter, angiography demonstrated patchy tumor staining. **(E)** Interventional angiography showed linear contrast agent passage within the original rectal stent, dilatation of the distal intestine, and contrast retention distal to the stent. **(F)** After repeat intestinal stent placement, contrast agent passed smoothly through the stent and was excreted.

Intestinal stent placement: The patient was placed in the supine position. A single-curve catheter together with a hydrophilic guidewire was inserted transanally and successfully passed through the stenotic segment. Angiography revealed marked dilatation of the distal intestine, contrast agent retention distal to the lesion, filling defects within the rectum, and rigid bowel wall (**Figure 2A**). The guidewire was exchanged for a stiff guidewire and advanced into the distal colon. Two intestinal stents were sequentially deployed along the guidewire, with slow release under precise fluoroscopic guidance. Postprocedural angiography demonstrated smooth passage of contrast agent through the stents (**Figure 2B**), and the patient began to pass flatus and stool after the procedure.

Regional intra-arterial infusion chemotherapy: Using the Seldinger technique, the right femoral artery was punctured, and a pigtail catheter was positioned at the abdominal aortic bifurcation for bilateral iliac angiography. Angiography revealed tumor staining in the pelvic cavity, with the lesion surrounding the rectal stents. The primary feeding artery was identified as a branch of the left internal iliac artery (**Figure 2C**). A 2.2F microcatheter was superselectively advanced into the feeding artery, and repeat angiography confirmed this branch as the dominant tumor-feeding vessel (**Figure 2D**). After securing the microcatheter in place, the patient was returned to the ward, and chemotherapeutic agents were slowly infused via the catheter using an arterial pump. The regimen consisted of raltitrexed 2 mg combined with oxaliplatin 100 mg, administered over 4 hours. This combination regimen was selected based on the patient’s tumor genetic testing results, which indicated sensitivity to both agents. As a thymidylate synthase inhibitor, raltitrexed exhibits comparable efficacy to 5-fluorouracil, with more convenient administration and a superior safety profile in elderly patients. Dosing was individually determined and appropriately reduced after comprehensive assessment of the patient’s age, body surface area, and hepatic and renal function. The patient tolerated treatment well, and no dose modification was performed during the course of therapy. Interventional treatment was repeated at 4-week intervals.

Deep hyperthermia: Deep hyperthermia was performed using a capacitive coupling system (WE2102-A, 40.68 MHz; Dalian Oriole Technology Co., Ltd., China). Recurrent rectal lesions were designated as the temperature monitoring target, with a 12-channel real-time temperature monitoring system employed to precisely maintain the target temperature at 41-43°C for 30 minutes per session (11). During treatment, Clinical parameter optimization, including the use of a surface coupling water balloon and adjustment of saline concentration, improved intratumoral temperature distribution, reduced superficial overheating, and ensured effective heating of deep pelvic tumors. Hyperthermia was administered as follows: once daily for 3 consecutive days post each interventional procedure, followed by once every 48–72 hours (2–3 sessions weekly). During treatment, vital signs, body temperature, and local skin condition were closely monitored. Power output was adjusted based on patient tolerance to avoid overheating. In case of any abnormality, timely management or treatment termination was implemented.

Bevacizumab targeted therapy: Complete blood count, coagulation profile, and liver and renal function were evaluated on day 3 after each interventional procedure. Concurrently, 600 mg of bevacizumab was administered intravenously according to Chinese Society of Clinical Oncology guidelines and tumor genotyping results, at 4-week intervals aligned with the interventional treatment cycles. Although the patient exhibited wild-type KRAS, cetuximab was not chosen given the superior safety profile of bevacizumab in elderly patients, characterized by lower risks of severe infusion reactions, acneiform rash, and electrolyte disturbances. The preexisting bladder fistula was stable and inactive at baseline; therefore, the potential benefits of vascular endothelial growth factor (VEGF) inhibition were considered to outweigh the corresponding risks. Close surveillance for fistula progression and hemorrhage was performed throughout treatment, with no related adverse events observed. Following completion of interventional therapy, maintenance bevacizumab was continued every 4 weeks, accompanied by tumor response assessment at 4-week intervals.

### Follow-up and outcomes

**Figure 3:**
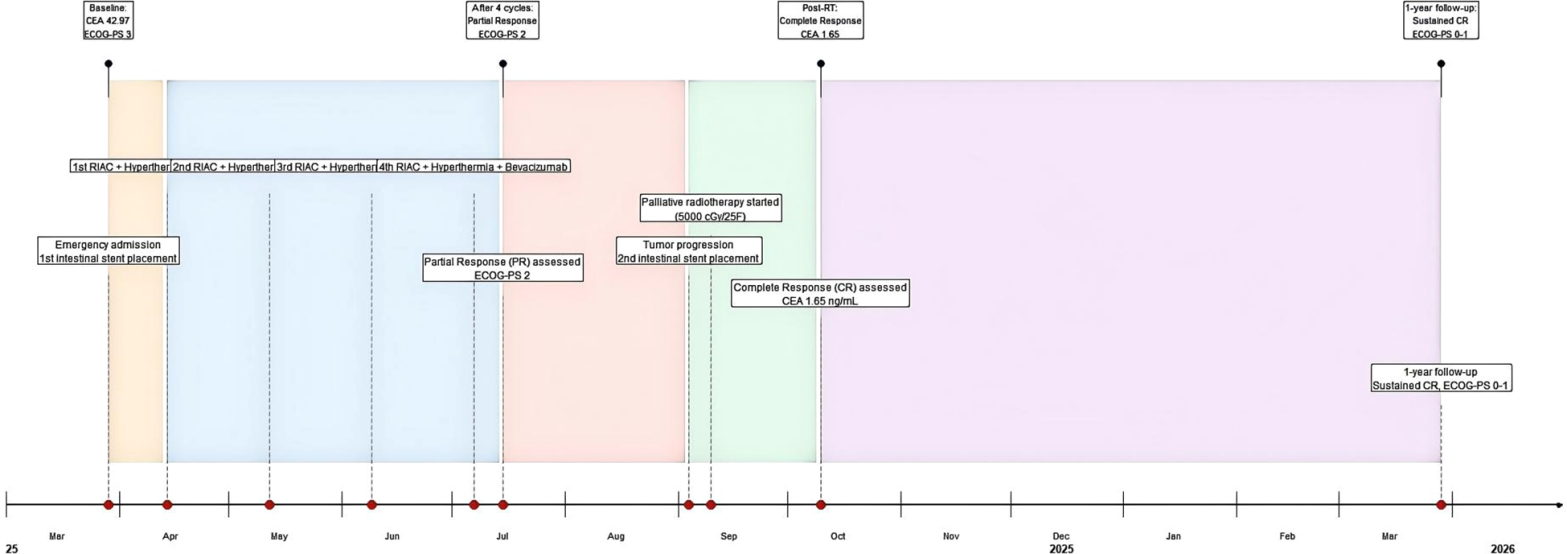
Timeline of clinical management and key outcomes: elderly patient (81 years) with recurrent rectal cancer after radical resection complicated by intestinal obstruction.

Routine hematological parameters, including complete blood count and liver and kidney function, were monitored before and after treatment at scheduled intervals; the detailed changes are presented in **Table 1**. Compared with baseline (**Figure 1B**), CT performed three weeks after the first interventional procedure showed a reduction in tumor size (**Figure 1C**), with evidence of liquefactive necrosis (**Figure 1D**). Three weeks after the third interventional procedure, the necrotic portion of the tumor was partially absorbed, with further reduction in tumor size and residual necrosis visible internally (**Figure 1E**). After completion of four cycles of interventional treatment, the tumor had markedly decreased in size, and the clinical response was evaluated as partial remission (PR) (**Figure 1F**). During the treatment course, recurrent intestinal obstruction occurred due to tumor in growth within the stent (**Figure 1G**), necessitating repeat intestinal stent placement (**Figures 2E, F**). Of note, the patient had achieved a partial response on imaging at the time of this event. Given that subsequent imaging demonstrated a persistent reduction in overall tumor burden, the intrastent tumor ingrowth was interpreted as local proliferation of residual viable tumor cells rather than systemic disease progression. This finding was interpreted not as a conventional adverse event, but rather as a mechanical complication associated with the local tumor–stent interface. The patient did not develop any systemic adverse reactions, including hematologic toxicity, severe fatigue, or organ dysfunction. Palliative radiotherapy was subsequently administered at a total dose of 5000 cGy in 25 fractions (200 cGy per fraction). One month after radiotherapy, the tumor had substantially regressed and disappeared, achieving a clinical complete remission (CR) (**Figure 1H**). Deep hyperthermia and bevacizumab targeted therapy were administered throughout the entire treatment course. One year after the initial interventional procedure, contrast-enhanced abdominal CT demonstrated stable disease with no evidence of tumor recurrence or metastasis (**Figure 1I**). CEA level had decreased to 1.65 ng/mL, and ECOG performance status improved to 0-1. Clinical evaluation confirmed sustained complete remission (CR) (**Figure 3**).

**Table 1:** Changes in hematological parameters, liver and kidney function, and CEA during treatment.

Time point	CEA (ng/mL)	ALT (U/L)	AST (U/L)	TBIL (μmol/L)	CREA (μmol/L)	UREA (mmol/L)	WBC (×10^9^/L)	HGB (g/L)	PLT (×10^9^/L)	NEUT (×10^9^/L)
Before first intestinal stent placement	/	19	17	30.5	82.40	7.77	8.94	112	343	11.60
4 days after first intestinal stent placement	/	37	33	26.1	76.80	6.02	9.86	104	315	11.75
Before first chemoperfusion	42.97	101	55	40.3	95.10	9.12	8.11	110	438	15.94
3 days after first chemoperfusion	3.92	60	44	21.5	85.3	7.30	7.95	83	387	6.81
Before second chemoperfusion	2.2	24	25	20.0	69.9	5.24	8.89	92	436	7.23
3 days after second chemoperfusion	5.13	20	27	33.1	71.70	5.86	7.29	102	435	9.33
Before third chemoperfusion	12.42	14	24	25.1	81	7.82	7.62	109	339	5.77
3 days after third chemoperfusion	13.82	15	23	37.8	86.20	7.63	9.97	100	313	8.71
Before fourth chemoperfusion	2.31	16	21	15.5	69.9	5.87	6.85	92	429	4.99
3 days after fourth chemoperfusion	2.37	19	26	23.2	64.9	5.76	5.91	82	321	4.59
Before second intestinal stent placement	7.74	20	23	32.9	87.30	8.87	9.01	106	407	9.68
1 month after radiotherapy	8.03	43	16	7.7	88.3	8.04	8.84	92	409	7.51
1 year after comprehensive treatment	1.65	36	27	16.8	91.70	10.29	7.38	100	383	6.10

## Discussion

Elderly patients with recurrent rectal cancer after radical resection complicated by intestinal obstruction have an extremely poor prognosis. The present case was further complicated by invasion of the bladder and left ureter, representing an even more challenging clinical scenario. Although emergent intestinal stent placement promptly relieved the obstruction and markedly improved the patient’s general condition, his initial performance status precluded tolerance to conventional chemoradiotherapy (12). The localized disease was advanced with adjacent organ invasion but without distant metastases. Based on genetic testing results and multidisciplinary team discussion, a relatively gentle combined local and systemic treatment strategy was formulated. After significant improvement in the patient’s general condition and evident tumor shrinkage, sequential local palliative radiotherapy was added, while deep hyperthermia combined with targeted therapy was administered throughout as a relatively gentle local-plus-systemic approach. Following this comprehensive treatment, the patient ultimately achieved long-term high-quality survival. Imaging follow-up demonstrated gradual tumor regression and eventual complete disappearance, with no evidence of recurrence or metastasis to date. Notably, this patient developed local recurrence less than four months after radical resection, despite having undergone low anterior resection with total mesorectal excision and achieving R0 resection. The early recurrence may be attributable to aggressive tumor biology, as evidenced by a high Ki-67 proliferation index, TP53 mutation, and negative PD-L1 expression. These features suggest that even after complete surgical resection, this patient remained at high risk for early recurrence, highlighting the need for effective salvage therapies in such high-risk populations. We attribute the favorable outcome to the following factors.

First, we consider that regional intra-arterial infusion chemotherapy(RIAC) as a precision tumor treatment may have contributed to the favorable outcome. The present patient received a 4-hour continuous infusion regimen. Clinical practice confirmed not only good patient comfort but also follow-up imaging evidence of liquefactive necrosis in the rectal tumor after RIAC, further demonstrating the effectiveness of this approach. Such effects cannot be replicated by systemic intravenous chemotherapy. Particularly for local tumors, RIAC can be considered a more precise chemotherapeutic modality. Furthermore, incorporating genetic testing results to guide selection of sensitive chemotherapeutic agents for RIAC achieves genuine precision oncology in both drug selection and treatment modality, resulting in not only favorable tumor control but also better patient tolerance.

Second, the integration of deep hyperthermia and bevacizumab throughout the treatment course may represent a key component of this comprehensive strategy and may have contributed to long-term stability in this patient. In this patient, deep hyperthermia was applied during RIAC, radiotherapy, targeted therapy, and treatment intervals to control local recurrent tumor. Clinical practice demonstrated no significant adverse effects while achieving specific killing and synergistic effects. Deep hyperthermia thus played a role in reducing toxicity and enhancing efficacy. We consider that hyperthermia may be considered a local treatment modality particularly suitable for patients with unresectable locally advanced rectal cancer.

As a sensitive targeted agent confirmed by tumor genetic testing, bevacizumab offers the advantage of fewer and milder adverse effects compared with cetuximab. As a systemic treatment, bevacizumab not only blocks angiogenesis by inhibiting VEGF but also induces a “tumor vascular normalization” effect (13, 14). Studies have confirmed that raltitrexed and oxaliplatin combined with bevacizumab synergistically control disease progression in advanced rectal cancer (15). Furthermore, deep hyperthermia can downregulate the expression of pro-angiogenic factors such as vascular endothelial growth factor (VEGF) and basic fibroblast growth factor (bFGF) in tumor cells and stromal cells, while upregulating angiogenesis inhibitors, thereby inhibiting tumor neovascularization (16, 17). Therefore, combination with deep hyperthermia further amplifies the effects of the aforementioned comprehensive treatment.

Radiotherapy remains an indispensable treatment modality for rectal cancer; however, the optimal timing of its application represents a challenging and critical decision point. In this patient, after four cycles of RIAC-based comprehensive treatment, with assessed significant tumor shrinkage and marked improvement in general condition, combined local palliative radiotherapy was administered. Throughout radiotherapy, deep hyperthermia was concurrently applied. Hyperthermia inhibits DNA repair enzyme activity, blocking post-radiotherapy DNA damage repair in tumor cells, and through complementary killing of tumor cell populations, thereby amplifying the therapeutic effect. After sequential radiotherapy, the tumor continued to shrink, ultimately achieving local complete remission. This timing strategy of “RIAC debulking followed by sequential radiotherapy” avoids the severe adverse reactions that may occur with radiotherapy under conditions of high tumor burden and poor patient status, while fully utilizing the optimal patient state created by comprehensive treatment. This approach is particularly suitable for elderly patients with locally advanced rectal cancer or postoperative recurrence who are intolerant to chemoradiotherapy, and warrants further summarization and promotion in clinical practice.

Finally, we must discuss the tumor immunological mechanisms underlying the clinical efficacy in this patient. The antitumor effect of RIAC combined with deep hyperthermia, radiotherapy, and targeted therapy in rectal cancer is not merely tumor cell killing, but rather a comprehensive effect achieved through multi-modality synergistic remodeling of the tumor immune microenvironment, activation of antitumor immune responses, and reversal of immunosuppression (18) (**Table 2**). Each treatment modality contributes to immune synergy across four dimensions: “induction of immunogenic cell death, disruption of immunosuppressive barriers, activation of immune cell function, and precision sensitization by targeted agents.” This converts the originally “cold tumor” (immunosuppressive type) into a “hot tumor” (immune-activated type), ultimately achieving immune-mediated killing and long-term control of the rectal tumor through multidimensional remodeling of the tumor immune microenvironment.

**Table 2:** Changes in immune function parameters during treatment.

Time point	IL-17A (pg/mL)	NK (%)	NKT (%)	CD4+/CD8+
Baseline (before first chemoperfusion)	5.42	11.74	6.04	1.13
After 2 cycles of HAIC combined with bevacizumab	6.85	14.20	8.50	1.21
After 4 cycles of HAIC combined with bevacizumab	4.30	16.80	10.90	1.38
1 month after radiotherapy	3.10	18.50	13.60	1.52
1 year after comprehensive treatment	2.77	15.40	12.20	1.50

The clinical course of this patient exemplifies three important stages of the antitumor immune mechanism. The first stage can be understood as bevacizumab targeted therapy achieving initial tumor vascular normalization, reducing secretion of immunosuppressive factors, and laying the immunological foundation for subsequent treatment (19). The second stage involved concurrent RIAC plus deep hyperthermia followed by sequential local radiotherapy. Simultaneous implementation of RIAC and deep hyperthermia, followed by sequential local radiotherapy (IMRT, total dose 45–50 Gy), synergistically induced robust immunogenic cell death, activated dendritic cell antigen presentation, and disrupted immunosuppressive barriers (20). The third stage consisted of targeted maintenance plus immune activation (21). After concurrent comprehensive sequential treatment, maintenance therapy with bevacizumab combined with hyperthermia was continued, persistently inhibiting tumor vasculature and immunosuppression, while utilizing the abscopal effects of radiotherapy and hyperthermia to achieve long-term activation of systemic antitumor immunity. Avoiding high-dose chemoradiotherapy that would cause extensive immune cell killing and ensuring immune cell survival and activation may be central to this combination strategy and potentially contributed to the favorable outcome.

## Data Availability

The original contributions presented in the study are included in the article/supplementary material. Further inquiries can be directed to the corresponding authors.
